# Otimizando o Tratamento para o Infarto Agudo do Miocárdio, um Esforço Contínuo

**DOI:** 10.36660/abc.20210907

**Published:** 2021-11-22

**Affiliations:** 1 Pontificia Universidad Catolica Cardiovascular Division Santiago Chile Pontificia Universidad Catolica - Cardiovascular Division, Santiago – Chile; 2 Universidad Catolica de Chile División Cardiovascular Santiago Chile División Cardiovascular, Universidad Catolica de Chile, Santiago – Chile

**Keywords:** Infarto do Miocárdio, Reperfusão Precoce, Educação e Treinamento

A reperfusão precoce com intervenção coronária percutânea primária (ICPP) ou trombólise (TL) diminuiu a morbimortalidade por infarto agudo do miocárdio (IAM) na América Latina e no mundo. Diretrizes nacionais e internacionais recomendam a reperfusão, por qualquer um dos métodos, de acordo com a disponibilidade no centro médico, para pacientes internados em até 12 horas desde o início dos sintomas de IAM.^1,2^

A ICPP tem se mostrado superior à TL, principalmente quando realizada em um centro de atendimento especializado com infraestrutura, equipe e experiência necessárias para garantir bons resultados e minimizar complicações.^3-5^ Entretanto, devido aos altos custos associados à implementação e desenvolvimento de programas de intervenção cardiovascular, a disponibilidade de centros com a capacidade de realizar essa intervenção varia por país e até mesmo por região dentro de um determinado país.

Evidências internacionais também indicam que a TL pré-hospitalar seguida de transferência em tempo hábil para um hospital de maior complexidade para um estudo invasivo pode igualar ou até mesmo exceder os resultados obtidos com a ICPP.^6^ Diante dos achados acima mencionados, alguns autores recomendam um estudo invasivo de rotina em pacientes trombolisados nas primeiras 24 horas após a reperfusão.^7^ A TL tem se mostrado particularmente eficaz se um trombolítico de última geração for administrado em tempo hábil, ou seja, nas primeiras 2-4 horas após o início dos sintomas.^8,9^ Estudos recentes têm demonstrado os benefícios da implementação de uma estratégia fármaco-invasiva, que é a TL com Tenecteplase em doses ajustadas ao peso e idade, seguida da transferência para um centro de ICP para realização de ICP de resgate ou eletiva, dependendo dos sinais de reperfusão positiva ou negativa.^9,10^ Médicos não-especializados costumam preferir a transferência para um centro de ICP, com grande demora, e consequente subutilização de trombolíticos.^11^

A implementação dessas estratégias tem sido realizada com sucesso na Europa e nos EUA e, sempre que possível, na América Latina, mas existem desigualdades persistentes em diferentes regiões. Grandes cidades, como São Paulo, representam um desafio, principalmente nos hospitais públicos, uma vez que a melhora no atendimento ao IM necessita de políticas públicas adequadas. Em primeiro lugar, as pessoas devem ser educadas sobre como reconhecer os sintomas e a necessidade de atendimento imediato nos serviços de emergência. Deve ser seguido um diagnóstico precoce feito pela equipe de saúde (médicos e enfermeiros), apoio do tele-eletrocardiograma para acelerar o diagnóstico e a implementação da melhor terapia de reperfusão disponível em cada centro. Isso implica usar TL quando as chances de transferência para um centro terciário irão atrasar o tempo para a reperfusão ideal.

No estudo realizado por Machado et al.,^12^ e publicado nesta edição dos Arquivos Brasileiros de Cardiologia, os autores relatam sua experiência com o treinamento de médicos e enfermeiros de serviços de emergência em cinco hospitais públicos que apresentam altas taxas de mortalidade por IAM no início do estudo e comparam resultados com dados de hospitais com pessoal não-treinado no seguimento de longo prazo. Os resultados obtidos são bastante impressionantes, pois alcançam uma redução significativa da mortalidade nos centros treinados.^12^ Certamente, essa é uma iniciativa que deve continuar com o passar do tempo e estendida a outros hospitais. A rotatividade do pessoal de saúde nos serviços de emergência representa um desafio adicional e, por isso, o treinamento deve ser continuado ao longo do tempo.

Essa experiência contou com o apoio da Sociedade de Cardiologia do Estado de São Paulo e da Secretaria de Saúde do Estado de São Paulo. Apoio adicional deve ser encontrado para agregar outras políticas, como educação, tele-eletrocardiograma e a construção de redes com centros terciários.^13^ Por último, mas não menos importante, seria conveniente que o tratamento do IM recebesse suporte das autoridades governamentais para facilitar a consulta no hospital disponível mais próximo. Essas políticas foram bem-sucedidas em outros países, onde alcançaram uma redução significativa na mortalidade por IM.^14^
