# Supplementary material for: Otimizando o Tratamento para o Infarto Agudo do Miocárdio, um Esforço Contínuo
Source: Arq Bras Cardiol. 2021 Nov 22;117(6):1079–80. [Article in Portuguese] doi: 10.36660/abc.20210907 (PMC8757167; doi:10.36660/abc.20210907)
Supplement: Translation

# Translation: Optimizing Treatment for Acute Myocardial Infarction, a Continuous Effort

**Affiliations:** 1 Santiago Chile Pontificia Universidad Catolica - Cardiovascular Division, Santiago – Chile; 2 Santiago Chile División Cardiovascular, Universidad Catolica de Chile, Santiago – Chile

**Keywords:** Myocardial Infarction, Early Reperfusion, Education and Training

Early reperfusion with primary percutaneous coronary intervention (PPCI) or thrombolysis (TL) has decreased the morbimortality from acute myocardial infarction (AMI) in Latin America and worldwide. National and international guidelines recommend reperfusion, using either method, according to its availability at the medical center, for patients admitted within 12 hours of AMI symptom onset.^1,2^

PPCI has been shown to be superior to TL, especially when performed at a specialized care center with the infrastructure, staff, and experience necessary to ensure good results and minimize complications.^3-5^ However, because of the high costs associated with implementing and developing cardiovascular intervention programs, the availability of centers with the capacity to perform this intervention varies by country and even by region within a given country.

International evidence also indicates that pre-hospital TL followed by timely transfer to a higher-complexity hospital for an invasive study can match or even exceed the results obtained with PPCI.^6^ Given the abovementioned findings, some authors recommend a routine invasive study of thrombolyzed patients within the first 24 hours after reperfusion.^7^ TL has been shown to be particularly effective if a latest-generation thrombolytic is administered in a timely fashion, that is, within the first 2-4 hours after symptom onset.^8,9^ Recent studies have shown the benefits of implementing a pharmacoinvasive strategy, that is TL with Tenecteplase at weight and age-adjusted doses, followed by transfer to a PCI center to perform rescue or elective PCI, depending on the signs of positive or negative reperfusion.^9,10^ Nonspecialized doctors usually prefer a transfer to a PCI center, with a long delay, and the consequent underuse of thrombolytics.^11^

The implementation of these strategies has been successfully performed in Europe and USA and whenever possible, in Latin America, but there are persistent inequities in different regions. Large cities, such as Sao Paulo, represent a challenge, particularly at public hospitals, since improving MI care needs adequate public policies. First, the people should be educated about how to recognize symptoms and the need for prompt care at the emergency services. This should be followed by an early diagnosis made by the health team (doctors and nurses), support of tele-electrocardiography to accelerate the diagnosis and the implementation of the best available reperfusion therapy at each center. This implies using TL when the chances for transfer to a tertiary center will delay the time for optimal reperfusion.

In the study performed by Machado et al.^12^ and published in this issue of *Arquivos Brasileiros de Cardiologia*, the authors report their experience with the training of doctors and nurses from emergency services in five public hospitals that exhibit high MI mortality rates at baseline, and compare results with data from hospitals with non-trained staff in the long-term follow up. The obtained results are quite impressive, since they achieve a significant reduction of mortality at the trained centers.^12^ Certainly, this is an initiative that should be continued over time and extended to other hospitals. Rotation of health personnel in emergency services represent an additional challenge and, for that reason, the training should be continued over time.

This experience was supported by the Society of Cardiology from the State of Sao Paulo and the Health Secretariat of Sao Paulo. Additional support should be found to add other policies such as education, tele-electrocardiography, and the building of networks with tertiary centers.^13^ Last, but not least, it would be convenient that treatment of acute MI could be supported by government authorities to facilitate consultation at the nearest available hospital. These policies have been successful in other countries, where they have achieved a significant reduction in MI mortality.^14^
